# Partial restoration of aromaticity of pentacene-5,7,12,14-tetrone on Cu(111)†

**DOI:** 10.1039/d3nr04848a

**Published:** 2024-01-09

**Authors:** Lorenz Brill, Jonas Brandhoff, Marco Gruenewald, Fabio Calcinelli, Oliver T. Hofmann, Roman Forker, Torsten Fritz

**Affiliations:** a Friedrich Schiller University Jena, Institute of Solid State Physics Helmholtzweg 5 07743 Jena Germany torsten.fritz@uni-jena.de +49 3641 9-47412 +49 3641 9-47400; b Graz University of Technology, Institute of Solid State Physics, NAWI Graz Petersgasse 16/II 8010 Graz Austria o.hofmann@tugraz.at +43 316 873 8466 +43 316 873-8964

## Abstract

The π-conjugation of organic molecules can be strongly influenced when functional groups are added to a molecule, for example when pentacene is converted into pentacene-5,7,12,14-tetrone (P4O) by substitution of four H-atoms with four O-atoms, leading to four C=O double bonds. In fact, although free P4O resembles the parent hydrocarbon pentacene structurally at a first glance, its electronic properties differ drastically and can be more accurately described by three benzene units connected *via* four carbonyl groups. If P4O is deposited onto Cu(111), the electronic interaction across the interface has previously been reported to fully restore the π-conjugation through a weakening of the C=O double bonds and a redistribution of electrons, both of which have been explained with the model of surface-induced aromatic stabilization. Here, we observe for the case of P4O on Cu(111) that the molecule does not exhibit full π-conjugation upon interaction with the surface, likely because of the special electronic nature of the hybridized P4O on Cu(111). Our results are derived from CO-functionalized noncontact atomic force microscopy measurements in combination with dispersion-corrected density functional theory calculations yielding bond lengths and molecular geometries. To characterize the aromaticity, we apply the harmonic oscillator model of aromaticity.

## Abstract 2

CO-AFM measurements and DFT calculations demonstrate that the aromaticity of pentacene-5,7,12,14-tetrone (P4O) is only partially restored upon interaction with the Cu(111) surface, contrasting the model of surface-induced aromatic stabilization.

## Graphical abstract

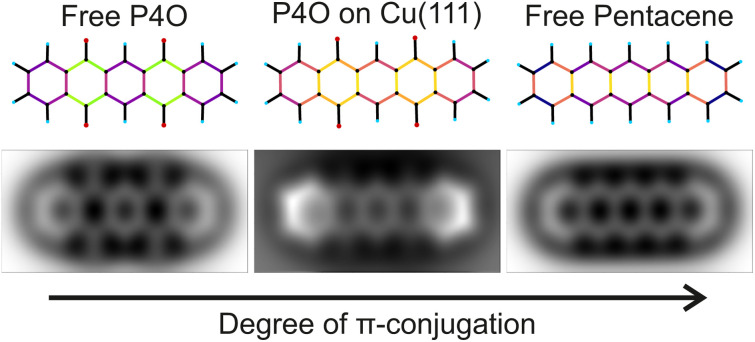

## 1. Introduction

**Fig. 1:**
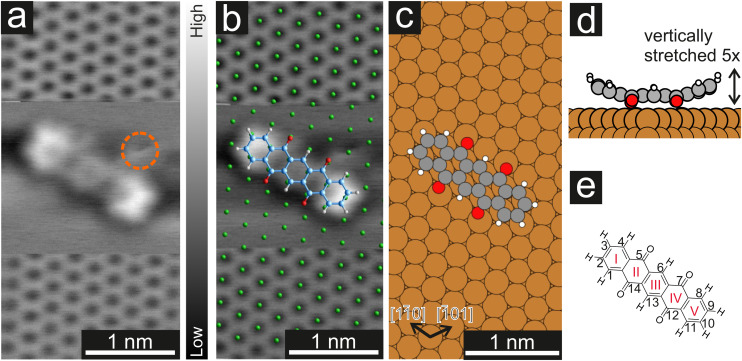
(a) CO-AFM image of the Cu(111) surface with atomic resolution and a P4O molecule on the surface. To image the molecule, the tip was retraced by 210 pm when recording the middle part of the image and re-approached for the bottom part. The orange circle marks an additional adatom/adsorbate that was present close to the molecule. To achieve a uniform contrast, a linewise average was subtracted from the raw data. (b) Distortion-corrected image overlayed with a model of the Cu(111) surface (green) and a model of the P4O molecule. The long molecular axis and the substrate [11̄0] direction form a 4(1)° angle. (c) Calculated adsorption geometry of P4O on Cu(111). Here, the angle between the long molecular axis and the [11̄0] substrate direction is 1°. (d) Side view of the calculated adsorption geometry. The total vertical deformation of the molecule is 60 pm which has been amplified by a factor of five to be more easily visible. (e) Structural formula of P4O and conventional numbering of the carbon atoms. The carbon rings are enumerated with red roman numerals.

Interfaces in layered organic (opto-)electronic devices fulfill a variety of essential functions, such as charge carrier injection or extraction, exciton separation, *etc*.^1–3^ The investigation of organic molecules on metal substrates aims at mimicking the contact plane between a functional film and an electrode.^4–6^ However, because of the complex nature of such systems, it is still challenging to account for the variety of interactions across the interface, including integer or fractional charge transfer, hybridization, molecular deformation, and the renormalization of electronic levels, amongst others.^7–12^ To capture several of those effects the so-called surface-induced aromatic stabilization (SIAS) model has been proposed.^13^ This model is particularly interesting for molecules whose π-electron system (which tends to delocalize in polycyclic aromatic hydrocarbons) is locally interrupted by functional groups, such as C=O substituting for C–H bonds. Upon interaction with a sufficiently reactive metal surface (*e.g.*, Cu(111)), the π-conjugation has previously been reported to be restored over the entire molecular backbone.^13^ The SIAS model has since been extended to many polycyclic hydrocarbons.^14–17^ In general, aromaticity is a property that is difficult to define.^18,19^ Multiple different criteria used to determine the aromaticity of organic molecules exist, focusing on varying properties of the molecules, *e.g.*, reactivity, geometric, electronic or magnetic properties.^20–26^ In this work, we use the harmonic oscillator model of aromaticity (HOMA).^23^ As a geometric criterion evaluating the C–C bond lengths in the molecule it is defined as1
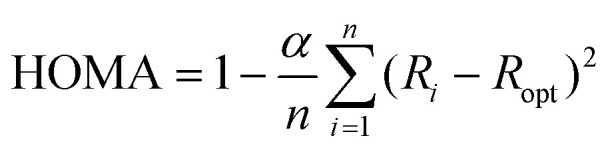
where *R*_*i*_ are all the bond lengths under consideration, *R*_opt_ is the energetically ideal bond length in a harmonic oscillator approximation, *n* is the number of bonds, and *α* is a normalization constant. A commonly used parameterization is: *R*_opt_ = 1.388 Å and *α* = 257.7 Å^−2^.^27^ By definition, the maximum HOMA value is 1 for benzene (which is referred to as a fully aromatic molecule), and the normalization constant *α* is chosen such that the HOMA value is 0 for the (hypothetical) non-resonant 1,3,5-cyclohexatriene. Values below 0 are possible, for example for antiaromatic molecules.^28^ Previously, the molecule pentacene-5,7,12,14-tetrone (P4O, CAS registry no.: 23912-79-0, see Fig. 1(e)) has been studied on coinage metal (111) surfaces as a model system for the effects of surface-induced aromatic stabilization.^13^ For free P4O molecules, the π-conjugation is disrupted by the four carbonyl C=O double bonds, and thus the aromaticity is effectively restricted to the first, third, and fifth benzene ring. According to SIAS, upon adsorption on the highly reactive Cu(111) surface, the C=O double bond weakens towards a single bond and the π-conjugation re-appears over the entire pentacene-type backbone.^13^ In this work however, we find a clear difference between P4O on Cu(111) and fully π-conjugated molecules like pentacene, indicating that additional effects not captured by the model of SIAS lead to a final state with only partially restored aromaticity. We use density functional theory (DFT) calculations to obtain the bond lengths required for the HOMA evaluation with high precision, and we perform CO-functionalized atomic force microscopy (CO-AFM) measurements to corroborate the theoretical results. Extracting the bond lengths directly from the experimental images is not feasible with sufficient precision because of tip-flexibility-induced imaging artifacts like bond elongation.^29–32^ Nonetheless, the excellent agreement of measured and simulated CO-AFM contrasts using the DFT results allows us to use the bond lengths calculated from DFT with confidence.

## 2. Methods

All DFT calculations were carried out with FHI-aims^33^ using the PBE functional^34^ and TS^surf^ vdW correction parameterized for metal surfaces.^35^ We used “tight” basis set defaults, as shipped with the code, throughout. All calculations were converged until the total energy and electron density reached thresholds of 10^−6^ eV and 10^−5^ e Å^−3^, respectively. For geometry optimizations we used the BFGS algorithm and relaxed the structure until the remaining forces were smaller than 10^−3^ eV Å^−1^. We sampled the Brillouin zone with 12 × 14 × 1 *k*-points for the monolayer and 5 × 5 × 1 *k*-points for the single molecule and used a Gaussian broadening of 0.01 eV for both. The calculations on Cu(111) used a slab of five Cu layers, where the top two layers were relaxed. The slabs were separated by 50 Å of vacuum. A dipole correction was introduced to electrostatically decouple the periodic replica in *z*-direction.^36^

The AFM simulations were carried out using the probe-particle model developed by P. Hapala *et al.*^37^ using standard parameters for CO tips *k*_lat_ = 0.25 N m^−1^, *Q* = −0.05 *e*, *Q*_dz^2^_ = 0.025 e Å^2^, *k*_tip_ = 540 000 N m^−1^ and the measured resonance frequency of the KolibriSensor *f*_res_ = 991 000 Hz.

All experiments were carried out at a base pressure of 10^−10^ mbar. The Cu(111) crystal was purchased from MaTecK GmbH (Jülich, Germany) and has a nominal purity of 99.999%. The crystal was cleaned by repeated Ar-sputtering (±45°, 700 eV, 4 μA cm^−2^) and annealing (600 °C), and the cleanliness verified with low-energy electron diffraction (LEED). The P4O molecules were purchased as a powder with a nominal purity of 95% from Alfa Aesar (Kandel, Germany) and further purified by temperature gradient sublimation. P4O was deposited onto the sample using molecular beam deposition for 30 s, while the substrate was kept at room temperature and the molecules were evaporated at a source temperature of 180 °C. Additional experiments with the substrate kept at cryogenic temperatures (≈ 20 K) during the deposition yielded no significant difference in the obtained adsorbate structures at very low coverage (not shown here). The LEED images were recorded using a commercial microchannel plate LEED from OCI Vacuum Microengineering Inc. (London, Ontario, Canada).

After the molecular deposition, the sample was precooled on a liquid-nitrogen-cooled shield before inserting it into a combined STM/AFM from SPECS Surface Nano Analysis GmbH (Berlin, Germany) operating at 4.5 K. The scanner uses a KolibriSensor,^38^ also manufactured by SPECS, which features a length extension resonator, leading to a much higher resonance frequency and spring constant than other commercially available resonators (such as the qPlus sensor^39^). Inside the STM/AFM, CO was deposited onto the sample by dosing CO into the chamber at a pressure of 10^−8^ mbar for around one minute and opening the optical ports of all the cryoshields during this time. The tip functionalization was performed by indenting the tip into the copper surface until a clean tip is achieved, then moving above a CO molecule and approaching the tip to the CO molecule at zero bias until the transfer can be seen in the Δ*f* response. Further details regarding the functionalization procedure are available in the ESI.†

## 3. Results and discussion

In a first step, we used CO-AFM to determine the lateral adsorption position of a single P4O molecule on Cu(111). For this purpose, we used an already established method, where the substrate is imaged with atomic resolution in the proximity of the molecule, so that the substrate lattice can then be extrapolated below the molecule.^40–43^ The results, as well as a comparison to the adsorption geometry determined by DFT, are depicted in Fig. 1. Since systematic errors are present in the raw data (for example shearing/stretching of the image due to drift), the measured atomic positions do not perfectly fit to a Cu(111) lattice. Therefore, the image distortions were corrected using LEEDLab^44^ before the copper lattice was overlayed and the molecular model was manually superimposed (details of the correction can be found in the ESI†). The resulting image (Fig. 1b) shows that the carbon rings are centered around substrate hollow sites, which is reminiscent of pentacene on Cu(111), where the same has been observed.^45^ The molecule is largely aligned to the [11̄0] substrate direction, but not perfectly so. Similarly, in the adsorption geometry determined by DFT calculations the molecule aligns with the [11̄0] substrate direction, with a very small deviation still present. In both cases, the misalignment is small (5° or less) and does not seem of fundamental importance. The ideal adsorption site determined by our DFT calculations is a bridge position, rather than a hollow site. Judging from the experimental resolution, it is difficult to precisely locate the molecular adsorption position due to the small distance between bridge and hollow sites. Further systematic errors in the measurements, such as the well-known bending effect of a CO-functionalized tip,^31,37^ likely contribute to the difference between experiments and calculations.

Furthermore, the side view of the calculated adsorption geometry (Fig. 1d) reveals a distinct bending of different parts of the P4O molecule towards or away from the surface. To scrutinize this vertical adsorption geometry we will now turn to CO-AFM images recorded at various tip–sample distances Δ*z*, where the closest approach is set as Δ*z* = 0 pm.

**Fig. 2:**
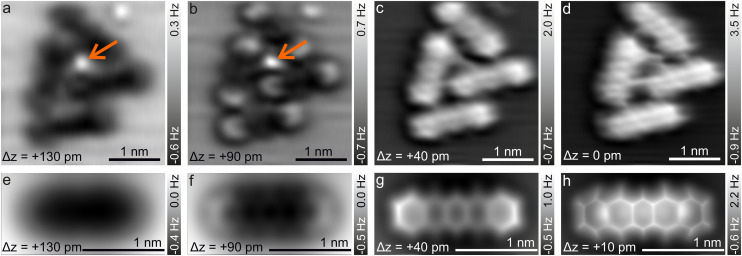
(a–d) 1D FFT-filtered CO-AFM measurements of a cluster of P4O molecules on Cu(111) around an additional adatom/adsorbate (orange arrow). The indicated Δ*z* values are relative, with the closest tip–sample distance being used as a reference point (Δ*z* = 0 pm). (e–h) Simulated CO-AFM images of a single P4O molecule on Cu(111). The simulations use the absolute tip–sample distance, which is inaccessible experimentally. Therefore, the best agreement with image (c) was used to fixate the Δ*z* values in the simulations. The Δ*z* values of the other panels were obtained with (c) and (g) as a reference point.

**Fig. 3:**
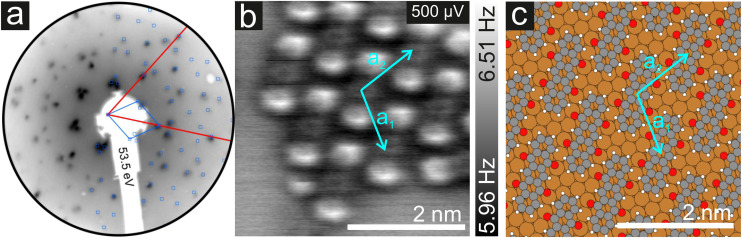
(a) Distortion-corrected LEED image of a single monolayer P4O (blue) on Cu(111) (red). (b) CO-AFM image of an island of P4O molecules on Cu(111) showing a similar structure to the one observed for a closed monolayer. The unit cell vectors determined by LEED are overlayed in cyan. (c) Structure obtained from the DFT calculations using the epitaxy matrix determined by LEED.

Fig. 2 shows the typical Δ*z* dependence of the CO-AFM contrast of P4O molecules on the Cu(111) surface. For large distances (Δ*z* = +130 pm), the molecules appear as dark ellipsoids due to the attractive van der Waals (vdW) forces. Upon decreasing Δ*z* to +90 pm, the ends of the molecules become visible first, accompanied by a faint signature of the third ring, while rings II and IV remain obscured. Rings II and IV become visible only after approaching the tip even further (Δ*z* = +40 pm), albeit slightly darker than the other rings. This difference finally becomes negligible once the tip is close enough (Δ*z* = 0 pm). A similar observation has been reported for pentacene on Cu(111), where the ends of the molecule are also brighter than the center because they are bent upwards.^41,46^ However, rings II, III, and IV of pentacene appear to be almost equally bright,^41,46^ whereas for P4O ring III is slightly brighter than rings II and IV as seen in Fig. 1(a), 2(c) and 3(b). To understand this contrast, we used the results of our DFT calculations to carry out CO-AFM simulations employing the probe-particle model developed by P. Hapala *et al.*^37^ In this model, the CO molecule is treated as a charged sphere and relaxed simultaneously in a harmonic spring potential describing the deflection from its equilibrium position and a force field consisting of a Lennard–Jones potential and an electrostatic potential generated by the sample. The resulting CO-AFM simulations are indeed consistent with the experimental data and corroborate the bent adsorption geometry obtained from the DFT calculations. Note that even if only the Lennard–Jones potential and the geometric data are considered in the simulation (*i.e.*, without taking electrostatic forces into account), the experimentally observed contrast is satisfactorily reproduced. Our DFT results show that the oxygen atoms pull the atoms C(5), C(7), C(12), and C(14) towards the surface, resulting in them being 4 pm lower compared to their neighboring carbon atoms. This height difference is already enough to result in the different appearances of rings II and IV compared to ring III. A detailed comparison between simulations with and without electrostatic forces (by virtue of the calculated electron density) as well as additional simulations demonstrating the impact of the height of the atoms C(5), C(7), C(12), and C(14) can be found in the ESI.†

In passing we note that the experimental data in Fig. 2 show a small molecular cluster because isolated P4O molecules on the Cu(111) terraces are very rare, even when depositing the molecules at cryogenic substrate temperatures (≈ 20 K). Instead, the molecules attach to substrate step edges and adatoms/adsorbates on the terraces whose exact nature is unknown, but is also not the focus of this work. We emphasize that the central adatom/adsorbate highlighted by the orange arrow is surrounded by three of the molecules imaged in Fig. 2, while the fourth molecule is further away. Yet, the experimentally obtained contrast does not differ significantly among those four molecules. In hindsight, this justifies our AFM simulations based on DFT data obtained on single molecules, *i.e.*, without additional adatoms.

As mentioned before, the contrast formation for the CO-AFM of P4O on Cu(111) seems to be mainly influenced by the geometric structure of the molecule, making it difficult to discern any electronic contributions. Additionally, the bent adsorption geometry influences apparent bond lengths in the AFM images. To further elucidate the influence of the copper surface on the electronic structure of the molecule, we use DFT calculations to compare P4O on Cu(111) to the free P4O molecule as well as fully π-conjugated molecules, namely free pentacene and a derivative of P4O, pentacene-5,7,12,14-tetraol (P4OL), where four additional hydrogen atoms are bonded to the oxygen atoms. To ensure comparability between the calculations, all of them were performed using the exact same method and convergence criteria. On the Cu(111) surface, we simulated both a single P4O molecule as well as a closed monolayer of P4O molecules. To obtain the appropriate unit cell for the monolayer, we took a low-energy electron diffraction (LEED) image of a sample with 1 ML P4O on Cu(111) (see Fig. 3(a)) and extracted the unit cell using LEEDLab after distortion-correcting the image with LEEDCal.^44^ This yields an essentially commensurate registry with the epitaxy matrix2
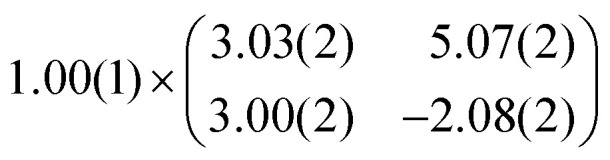
and both lattice vectors 
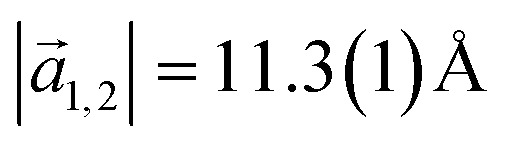
 with an angle of 107.8(2)°. The errors of the single matrix elements are the statistical uncertainties during the numerical fitting procedure (1*σ*), while the error of the pre-factor refers to uncertainties in the calibration factor of the device, influencing all matrix elements likewise. As can be seen in Fig. 3(b), these lattice vectors are similar to the structure of a P4O island observed with CO-AFM. The small differences (with 
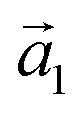
 being 0.6 Å too short and 
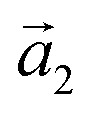
 being 0.4 Å too long in the CO-AFM measurement) can be attributed to drift in the AFM scan. With regard to the long measurement duration of 23 min for this image these deviations from the lattice vectors obtained with LEED are deemed acceptable.

For the DFT structure optimizations of the close packed monolayer, we used the epitaxy matrix extracted from the LEED data and obtained the structure shown in Fig. 3(c). The adsorption site is unchanged compared to the single molecule, it remains a bridge position. We note that this structure differs from previously published structural data of P4O on Cu(111), where the lattice constants were determined exclusively from STM images.^13^

**Fig. 4:**
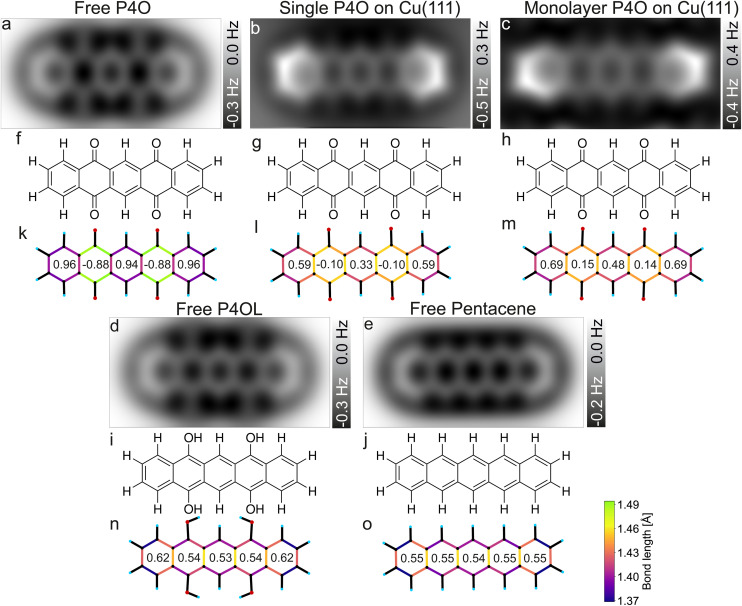
Simulated CO-AFM images of P4O and related molecules (a–e) as well as their structural formulas (f–j) and bond lengths from DFT (k–o). The numbers in (k–o) indicate the aromaticity of each carbon ring according to the HOMA, see eqn (1). The C–C bonds in (k–o) are color coded according to their lengths.

Previously, a full aromatic stabilization of the P4O molecule by the Cu(111) surface was reported,^13^ meaning that the originally interrupted π-electron system should extend over the whole P4O molecule upon interaction with the copper surface. This aromatic stabilization is described as an effective conversion of the P4O molecule into P4OL.^13^ The additional hydrogen atoms of the P4OL molecule transform the C=O double bond into a single bond, thereby mimicking the reported effect of the Cu(111) surface on the adsorbed P4O. To determine the aromaticity of the different molecules, we use the harmonic oscillator model of aromaticity (HOMA).^23^ We emphasize that we determine the HOMA value of each individual carbon ring and evaluate the degree of π-conjugation by the *variance* of values between the rings, while we do not discuss the *absolute* HOMA values. For a fully π-conjugated molecule, all rings should have a similar HOMA value, whereas if the π-conjugation is interrupted, the rings should differ significantly. Additionally, we simulated the AFM contrast for all molecules by the probe-particle model mentioned above, using both the geometry and the electron densities obtained from our DFT calculations. All of these results are displayed in Fig. 4.

Pentacene and P4OL are similar to each other, both in aromaticity and in the AFM contrast, because only single bonds are present outside of the carbon rings. Both molecules have very similar HOMA values across the entire backbone, as is expected for a fully π-conjugated molecule. The AFM contrast is mostly uniform over the molecule, with the C(1)–C(2)–C(3)–C(4) as well as the C(8)–C(9)–C(10)–C(11) atoms (compare Fig. 1(e)) and corresponding bonds being slightly brighter, mainly because of the different vdW background. At both ends of the molecule, the total vdW force is smaller, leading to an increase in frequency shift.^47^ For the P4OL molecule the oxygen atoms are also visible, although less pronounced than the carbon atoms, while the rings with oxygen atoms attached appear slightly darker than the central ring, mainly because of distortions introduced by the nearby oxygen atoms. In contrast, rings II and IV of the free P4O molecule appear noticeably darker than rings I, III, and V, which is readily apparent in the AFM simulation. This difference in brightness stems mainly from the significantly elongated C–C bonds in rings II and IV, due to the influence of the oxygen atoms being attached there, which in turn gives rise to a decreased aromaticity in these rings according to the HOMA model. The difference in bond lengths (not discernible in Fig. 4(a)) only becomes visible when the CO-tip is even closer and bond-sharpening^37^ plays a more pronounced role. Longer bonds appearing darker when the tip is far enough away from the molecule to avoid strong bond sharpening matches very well with the previously observed contrasts of hexa-*peri*-hexabenzocoronene (HBC, CAS registry no.: 190-24-9) on Cu(111).^31^

As mentioned before, free pentacene and P4OL are fully π-conjugated, whereas the π-conjugation is clearly interrupted for free P4O, which is also easily seen in the HOMA values of rings II and IV of the free P4O molecule. For P4O on Cu(111), both the single molecule as well as the monolayer, we obtain a result in between these two extremes: there is still a clear difference in the HOMA values of rings II and IV compared to the other three rings, yet this difference is not as large as in free P4O. Thus, the variance of the HOMA values along the P4O molecule decreases (meaning that the π-conjugation increases) when adsorbed on Cu(111). Importantly though, a fully π-conjugated electron system is not obtained and therefore P4O does not reach a fully aromatic state upon interaction with the copper surface.

While the HOMA values depend quadratically on bond length differences, the bond lengths themselves can be visualized linearly using a color code, for example. This does therefore not depend on eqn (1) and the parameterization of the HOMA model. The qualitative difference between the fully π-conjugated molecules and P4O adsorbed on Cu(111) can be directly discerned from the color-coded bond lengths of the molecules in Fig. 4(k–o). From a comparison of the DFT results for the single molecule and the close packed monolayer we deduce that the system seems to be dominated by the substrate–molecule interaction, while molecule–molecule interactions seem to play only a minor role. The molecular geometry changes only in minor ways from the single molecule to the closed monolayer, as indicated by the similar bond lengths and HOMA values. The HOMA values increase slightly in the monolayer, which might be because of intermolecular interactions, but could also result from the limited accuracy of the employed PBE functional. Nevertheless, the molecule is still clearly not fully π-conjugated, even in the closed monolayer.

For the bond lengths between the carbon and oxygen atoms a similar trend can be observed as for the π-conjugation: for P4O on Cu(111) that bond length is 1.308 Å for the single molecule and 1.307 Å in the monolayer, thereby corresponding to approximately the average of free P4O (1.227 Å, C=O double bond) and free P4OL (1.372 Å, C–O single bond). As stated above, this is consistent with a weakening of the C=O double bonds of P4O upon interaction with the Cu surface; yet, this yields atomic distances which are not in agreement with neither the standard values of single nor double C–O bonds but rather something in between. Thus, the analysis of the C–C and C–O bonds suggests that the interaction with the Cu(111) surfaces restores the aromaticity of P4O only partially, but does not reach the character of the fully π-conjugated pentacene or P4OL.

## 4. Conclusion

In conclusion, we use first principles calculations in conjunction with CO-AFM measurements and simulations to obtain reliable molecular geometries. Both methods capture the bent geometry of the molecules well and are in excellent agreement with each other. Based on the geometry, we determine the aromaticity of the single carbon rings of the P4O molecule on the Cu(111) surface for both a single molecule as well as a closed monolayer in comparison to free P4O and to the fully π-conjugated free molecules P4OL and pentacene by means of the HOMA model. While the aromaticity of P4O increases and the C=O bonds weaken upon interaction with the Cu(111) surface, the final state is not a fully aromatic acene-like character. Therefore, we find that the surface-induced aromatic stabilization occurs to a lesser extent than originally proposed for this system. This means that while the model can be of great help, it should be considered carefully for very strongly interacting systems, since at least for the case of P4O on Cu(111), additional effects uncaptured by the model seem to influence the adsorption process.

## Conflicts of interest

There are no conflicts to declare.

## Supplementary Material

NR-016-D3NR04848A-s001
