# Supplementary material for: Partial restoration of aromaticity of pentacene-5,7,12,14-tetrone on Cu(111)
Source: Nanoscale. 2024 Jan 9;16(5):2654–61. doi: 10.1039/d3nr04848a (PMC10832359; doi:10.1039/d3nr04848a)
Supplement: NR-016-D3NR04848A-s001 [file NR-016-D3NR04848A-s001.pdf]

Electronic Supplementary Material (ESI) for Nanoscale.
This journal is © The Royal Society of Chemistry 2024

Electronic Supplementary Information: Partial Restoration of Aromaticity of Pentacene-5,7,12,14-tetrone on Cu(111)

Lorenz Brill,† Jonas Brandhoff,† Marco Gruenewald,† Fabio Calcinelli,‡ Oliver T. Hofmann,*,‡ Roman Forker,† and Torsten Fritz*,†

†*Friedrich Schiller University Jena, Institute of Solid State Physics, Helmholtzweg 5, 07743 Jena, Germany*

‡*Graz University of Technology, Institute of Solid State Physics, NAWI Graz, Petersgasse 16/II, 8010 Graz, Austria*

E-mail: o.hofmann@tugraz.at; torsten.fritz@uni-jena.de

CO functionalization

First, the tip was deliberately decorated with copper atoms by indentation into the Cu(111) surface. The high reactivity of copper enables an easier pickup than with a bare tungsten tip. Afterwards, the tip condition was checked by means of STM scans. Once the tip is satisfactorily prepared, a CO molecule is located with an STM scan. Then, the tip is moved above the molecule (setpoint: 10 mV, 50 pA), the z-feedback is turned off, and the bias is reduced to zero. Now, the tip is approached manually in steps of 10 pm until a sudden jump in the current and frequency shift can be observed, an example can be seen in Figure S1(a). This usually happens at currents between 1 nA and 10 nA. Once the jump occurred, the tip is retracted manually with the same step size. As a preliminary criterion, the CO transfer

to the tip is deemed successful if the frequency shift after the retraction has changed by at least 500 mHz with respect to the unfunctionalized tip.

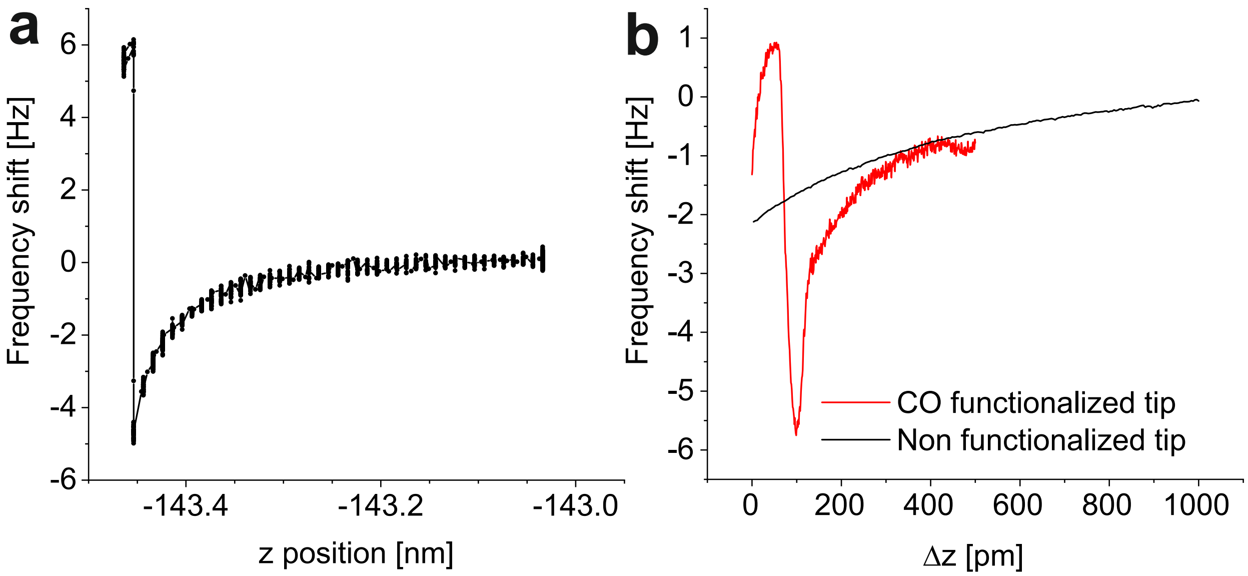

Figure S1: (a) Sudden jump in frequency shift upon picking up a CO molecule. There are multiple values for every z-position because of the manual approach. (b) Comparison between two frequency shift curves (depending on the distance Δz), with and without tip functionalization.

There are several ways to check the condition of a functionalized tip. (i) With a functionalized tip the Δf versus z curve should be much steeper than for unfunctionalized tips (cf. Figure S1(b)) at tip-sample distances which lead to currents below the amplifier saturation of 10 nA. In contrast, an unfunctionalized tip shows a steady decrease of Δf until the tip is so close to the surface that the saturation current of 10 nA is reached. (ii) With the functionalized tip it is possible to record atomically resolved images of the surface (cf. Figure S3). To do so, the tip needs to be approached until a current of around 300 pA to 500 pA at nominally 0 V is reached (note that our Nanonis electronics has a small voltage offset of $\approx -250\,\mu$V). CO-AFM scans were performed very slowly (i.e., using scan speeds $< 2\,\mathrm{nm\,s^{-1}}$) to obtain reasonable images. In our experiments, constant-height scans were generally preferred, since the z-feedback loop is avoided. If the tip functionalization was unsuccessful, no atomic resolution could be achieved, no matter how close the tip was approached. (iii) If another CO molecule is present on the Cu(111) surface, one can perform

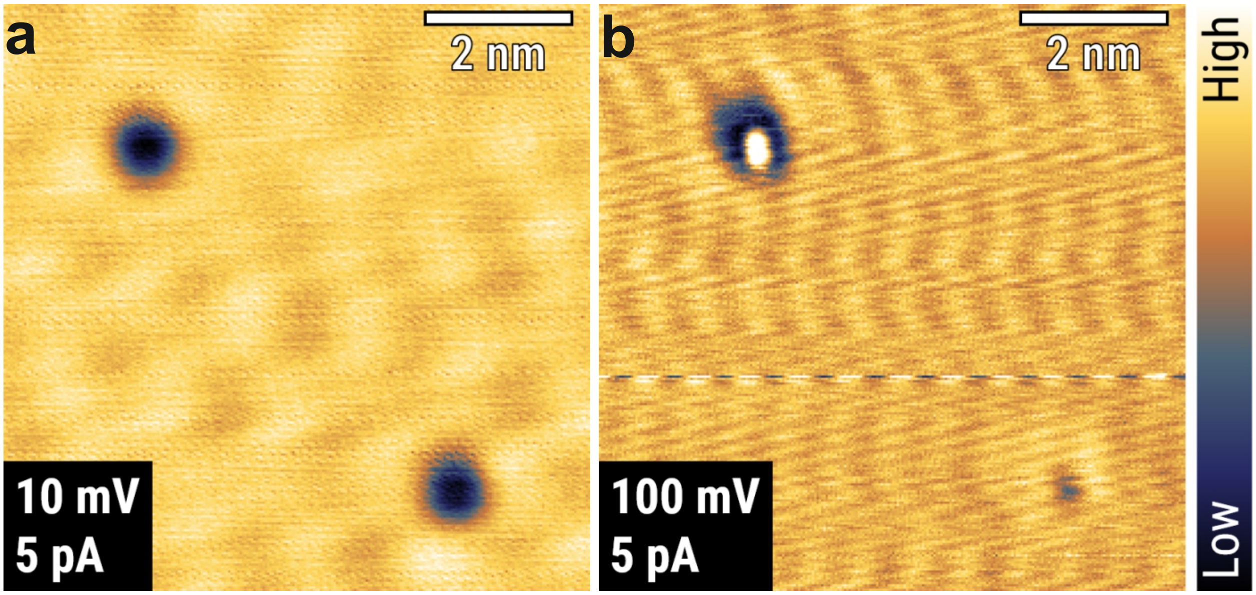

Figure S2: STM images acquired before (a) and after (b) successful tip functionalization. The contrast signature of the CO molecule in the bottom right is gone in (b), while the CO molecule in the top left displays a bright spot in its center.

another STM scan over this CO molecule (and ideally also over the site of the preceding CO pickup). These CO-STM scans should preferably be performed with a low current, a bias of 10 mV to 100 mV, and a scan speed of $10\,\mathrm{nm\,s^{-1}}$ to $20\,\mathrm{nm\,s^{-1}}$ to avoid destroying the functionalization. The site of the CO pickup should now be devoid of the CO molecule while the other CO molecules present show a bright spot in their center.[1,2] An example can be seen in Figure S2. If the bright spot is off center, it indicates that the CO molecule at the tip has been adsorbed at an angle. This is typically not problematic, but can lead to noticeably asymmetric images for large tilt angles.

After a successful functionalization, it is possible to swap the sample without destroying the functionalization. To do so, the tip was retracted as far as possible ($\approx$ 2 cm) before inserting the new sample, which has been precooled on the LN_2 shield. Afterwards, the microscope was left to cool down again to under 10 K before reapproaching the tip. To avoid an accidental tip crash, the approach was carried out more slowly compared to approaches with a bare metal tip. Figure S3 shows an atomically resolved Ag(111) surface, where the functionalization was carried out on a Cu(111) crystal and preserved while swapping to

the Ag(111) crystal. Using the two atomically resolved images and determining the lattice constants *via* fast fourier transform yields 2.51(2) Å for Cu(111) and 2.73(3) Å for Ag(111), in good agreement with the expected values of 2.55 Å[3] and 2.88 Å[4] respectively.

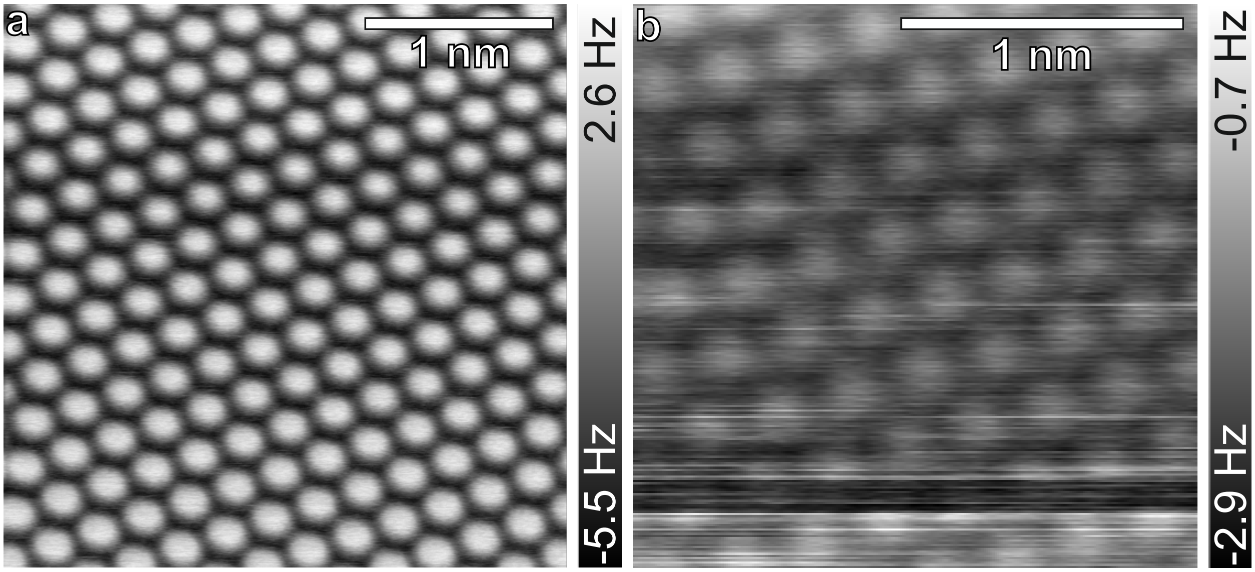

Figure S3: Atomically resolved surfaces of (a) Cu(111) and (b) Ag(111). Both images display raw data, recorded at 0 V, acquisition speed: (a) $1\,\mathrm{nm\,s^{-1}}$, (b) $2\,\mathrm{nm\,s^{-1}}$. To record image (b), the tip was functionalized on the Cu(111) crystal before swapping the sample to the Ag(111) crystal.

Image correction using LEEDLab

When using the raw data of Figure 1a in the main text, the measured positions of the copper atoms deviate somewhat from a theoretical copper lattice because of the piezo-induced image distortions and drift present in the measurement. To combat this, we used LEEDLab, a homemade software that contains fitting routines for spot-like objects in a 2D pixel map.[5] When applied to an atomically resolved CO-AFM image of a Cu(111) surface, LEEDLab automatically determines the positions of the copper atoms by fitting 2D Gaussian intensity distributions to each feature individually. Then, the residuals compared to an ideal copper lattice are calculated, which are stored in a vector field and represent the image distortions. These residuals are then used to remove the distortions from the image appropriately, so that

the atom positions in the corrected image accurately coincide with the expected positions.

Comparison of CO-AFM simulations

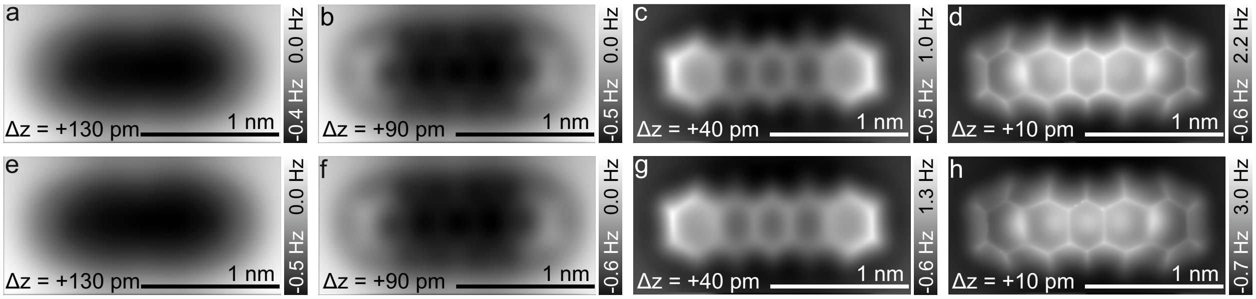

Figure S4: (a-d): CO-AFM simulations of single P4O molecules on Cu(111) considering full interaction. (e-h): Simulations using only the atom positions and Lennard-Jones interaction.

Figure S4 shows a comparison between CO-AFM simulations for P4O single molecules on Cu(111), where one set of simulations takes the full electrostatic interaction between tip and sample into account, whereas the other set uses only the atom positions and Lennard-Jones interaction. No significant difference is visible between the two sets of images. In particular, the different appearance of the central ring compared to rings II and IV is present in both sets of images, especially in panels (c) and (g). This means that the contrast stems mainly from the geometric form of the molecule, with the electronic structure playing only a very minor role.

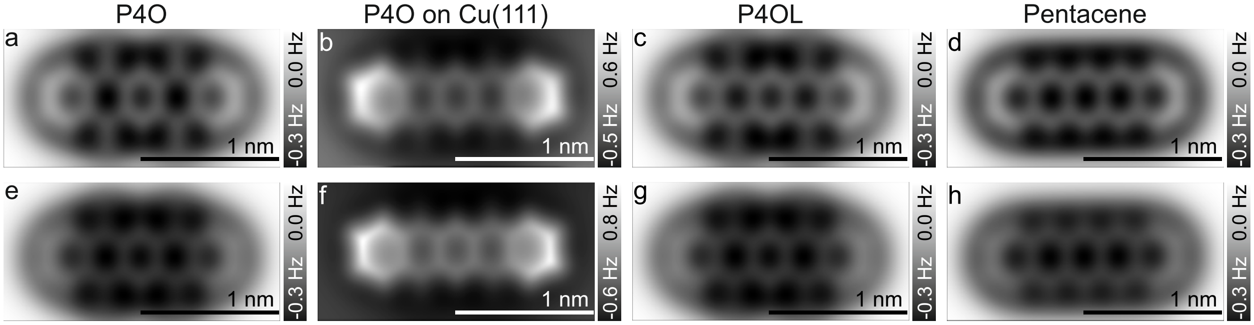

Figure S5: Simulations of different free molecules. (a-d): considering full interaction. (e-h): using only the atom positions and Lennard-Jones interaction. For comparison, the corresponding simulations of single P4O molecules on Cu(111) are also shown.

Figure S5 shows a similar CO-AFM simulation of different free molecules compared to P4O on Cu(111). Once again, the differences between the two simulation sets are subtle. The main difference is that the oxygen atoms are slightly more pronounced in the full interaction simulation, because of the repulsive electrostatic interaction between the oxygen atoms and the CO molecule. Still, all features are reproduced when considering Lennard-Jones interactions only.

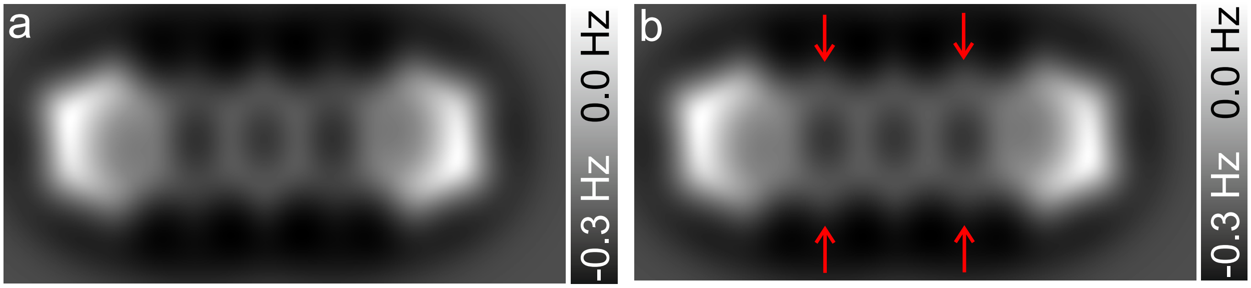

Figure S6: (a) CO-AFM simulation of P4O on Cu(111) using only Lennard-Jones interactions. (b) Same as in (a), but the four carbon atoms marked by the red arrows have been artificially lifted away from the surface by 4 pm, resulting in a changed contrast of rings II and IV.

The impact of the height of the C(5), C(7), C(12), and C(14) atoms on the uniformity of the simulated CO-AFM contrast is depicted in Figure S6. Figure S6(a) simply shows the CO-AFM contrast of a P4O molecule on Cu(111) (neglecting electrostatic interactions). In Figure S6(b), the C(5), C(7), C(12), and C(14) atoms were artificially lifted from the Cu(111) surface by 4 pm, therefore no longer representing an energetically optimal conformation. This change in height results in the rings II, III, and IV showing a nearly uniform contrast in the simulation, while a clear difference between these rings can be seen in the simulation using the energetically optimal conformation. Therefore, this height difference is sufficient to explain the dissimilarities in contrast between the rings.

Error estimation for HOMA values

Since the HOMA considers only the bond lengths, they need to be known with high precision to get a reliable index of aromaticity. Using the definition of HOMA:

$$\text{HOMA} = 1 - \frac{\alpha}{n}\sum_{i=1}^{n}(R_i - R_{\text{opt}})^2 \tag{S1}$$

and linear error regression, one obtains:

$$|\Delta\text{HOMA}| = \frac{\alpha}{n}\sum_{i=1}^{n} 2 \cdot |R_i - R_{\text{opt}}| \cdot |\Delta R_i| \tag{S2}$$

Here, we treat α and R_{opt} as invariable parameters, because they were *defined* within the HOMA model with the aim to yield specific values for two reference molecules (see main text). Therefore, both parameters are *not* included in our estimation of the uncertainties of the determined HOMA values. Because of the quadratic nature of the HOMA formula, a carbon ring with six benzene-like bond lengths will have an error of zero irrespective of the bond length uncertainty when using equation (S2). For each carbon hexagon in a free

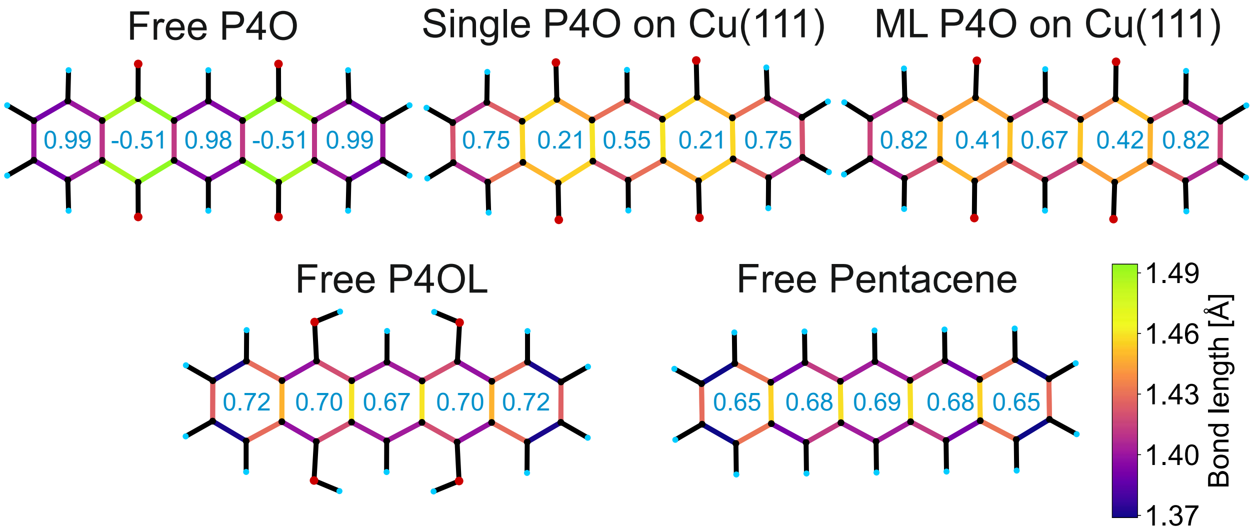

Figure S7: HOMA values of the molecules discussed in the main text where all bond lengths are artificially decreased by 1 pm. While all HOMA values increase, the variance of values across the molecules is robust with respect to this change.

pentacene molecule, assuming a typical uncertainty of $|\Delta R_i| = 1\,\text{pm}$ for all bond lengths leads to a HOMA uncertainty of 0.20 while the HOMA value is 0.55. This means that a bond length uncertainty of just 3 pm already leads to an uncertainty that is *larger* than the actual HOMA value, which would jeopardize any discussion of the HOMA values and their variations. Therefore, the bond length precision required for reliable HOMA analysis is not reachable with CO-AFM measurements, because bond elongation[6,7] and the bending of the molecule contribute to a much higher experimental uncertainty than 1 pm. Note that state-of-the-art CO-AFM measurements indicate experimental bond length uncertainties in the order of 20 pm in addition to the bond elongation.[6] Therefore, we turned to DFT calculations to obtain the bond lengths with higher precision. Nonetheless, the CO-AFM measurements are necessary to corroborate the DFT calculations and to use the computed results with confidence. Giving a reliable error estimate for bond lengths obtained by DFT is difficult, especially for organic molecules on metal surfaces, where many different types of bonds are present. To check that our evaluation of aromaticity is robust with respect to small errors, we performed the same evaluation with all bond lengths artificially elongated or compressed by 1 pm, with the results of the compressed case shown in Figure S7. While the HOMA values can change significantly, especially when far away from 1, the trend in *variance* of the values stays the same: Fully π-conjugated molecules like P4OL and pentacene have almost the same value within each carbon ring, whereas rings II and IV in free P4O have a very different value from rings I, III, and V. P4O on Cu(111), both single molecules and the closed monolayer, is between these two extremes. This shows that our evaluation of aromaticity is not significantly impacted by small systematic errors in the bond length determination that could stem from the DFT calculations.

References

1. Bartels, L.; Meyer, G.; Rieder, K.-H.; Velic, D.; Knoesel, E.; Hotzel, A.; Wolf, M.; Ertl, G. Dynamics of Electron-Induced Manipulation of Individual CO Molecules on Cu(111). *Phys. Rev. Lett.* **1998**, *80*, 2004–2007.

2. Gross, L.; Mohn, F.; Moll, N.; Liljeroth, P.; Meyer, G. The Chemical Structure of a Molecule Resolved by Atomic Force Microscopy. *Science* **2009**, *325*, 1110–1114.

3. Lu, X.-G.; Chen, Q. A CALPHAD Helmholtz energy approach to calculate thermodynamic and thermophysical properties of fcc Cu. *Philos. Mag.* **2009**, *89*, 2167–2194.

4. Guo, X.; Xu, Z.-F.; Lu, X.-G. A CALPHAD Helmholtz Energy Approach to Assess the Thermodynamic and Thermophysical Properties of fcc Ag. 2nd Annual International Conference on Advanced Material Engineering (AME 2016). 2016.

5. Fritz & Sojka GbR LEEDLab and LEEDCal. `https://fritz-sojka-gbr.de/`, `https://fritz-sojka-gbr.de/`.

6. Gross, L.; Mohn, F.; Moll, N.; Schuler, B.; Criado, A.; Guitián, E.; Peña, D.; Gourdon, A.; Meyer, G. Bond-Order Discrimination by Atomic Force Microscopy. *Science* **2012**, *337*, 1326–1329.

7. Jelínek, P. High Resolution SPM Imaging of Organic Molecules with Functionalized Tips. *J. Phys.: Condens. Matter* **2017**, *29*, 343002.
